# 宣威烟煤粉尘诱发树鼩支气管粘膜上皮改变的研究

**DOI:** 10.3779/j.issn.1009-3419.2015.08.01

**Published:** 2015-08-20

**Authors:** 小波 陈, 猛 贺, 光剑 李, 永春 周, 光强 赵, 玉洁 雷, 凯云 杨, 琳玮 田, 云超 黄

**Affiliations:** 1 650118 昆明，昆明医科大学第三附属医院（云南省肿瘤医院）胸外科一病区 Department of Cardiothoracic Surgery, the Third Affiliated Hospital of Kunming Medical University (Tumor Hospital of Yunnan Province), Kunming 650118, China; 2 276800 日照，山东省日照市人民医院胸心外科 Department of Cardiothoracic Surgery, Rizhao People's Hospital, Rizhao 276800, China; 3 518057 深圳，香港中文大学深圳研究院 Shenzhen Research Institute of Chinese University Hong Kong, Shenzhen 518057, China

**Keywords:** 树鼩, 宣威烟煤粉尘, 支气管上皮, 肺肿瘤, Tree shrews, Xuanwei bituminous coal dust, Bronchial epithelium, Lung neoplasms

## Abstract

**背景与目的:**

肺癌在许多国家和地区已成为发病率和死亡率最高的恶性肿瘤，建立科学合适的肺癌动物模型，用以模拟出与人类肺癌的病因、发病机制、发展过程相似的动物模型是亟待解决的问题。通过宣威烟煤粉尘PM10（particulate matter with diameters of 10 μm or less, PM10）对树鼩支气管上皮的影响，探索建立宣威烟煤粉尘致肺癌模型的可行性。

**方法:**

健康成年树鼩，切开颈部皮肤，充分暴露甲状软骨，于甲状软骨上方薄弱处，采用特制灌注针行穿刺的方法进行气管内试剂灌注。定期行X线检查，观察肺部影像学改变，处死动物行肺组织病理检查，观察灌注后支气管上皮改变情况。

**结果:**

烟尘处理组树鼩灌注药物后1周内开始死亡，空白对照组、溶剂对照组树鼩灌注后至实验结束无异常死亡。定期处死树鼩行肺组织HE染色切片病理检查，空白对照组及溶剂对照组无明显病理改变，烟尘处理组树鼩肺组织可见支气管粘膜上皮过度增生-鳞状化生-不典型增生-早期浸润癌的病理变化过程。

**结论:**

宣威烟煤粉尘可以导致树鼩支气管上皮出现支气管粘膜上皮过度增生-鳞状化生-不典型增生-早期浸润癌的病理变化，应用宣威烟煤粉尘PM10行气管内灌注可以诱发树鼩肺癌。可以建立肺癌模型。

## Abstract (en)

**Background and objective:**

Lung cancer is the type of cancer with the highest incidence and mortality in numerous countries and regions. Establishing an appropriate animal model that can be used to simulate lung cancer etiology, pathogenesis, and similar processes, is urgent. We explore the feasibility of establishing a lung cancer model induced by Xuanwei bituminous coal dust PM10 (particulate matter with diameters of 10 μm or less), which affects bronchial epithelium of tree shrews.

**Methods:**

The neck skin of adult tree shrews is dissected, and the thyroid cartilage is fully exposed. Subsequently, the weak part at the top of the thyroid cartilage is treated with intratracheal agents by perfusion via a special infusion needle puncture method. Regular X-ray examination and lung tissue biopsy were performed on the sacrificed animals to observe changes in pulmonary imaging and bronchial epithelial cells after perfusion.

**Results:**

The tree shrews of the experimental group (exposed to bituminous coal dust) died in a week, whereas no animal died until the end of the experiment in the blank control and the solvent control groups. Sections of lung tissue biopsy of the regularly killed tree shrews were stained with hematoxylin and eosin. The lung tissues of tree shrews in the experimental group showed changes caused by bronchial epithelial hyperplasia, squamous metaplasia, dysplasia, and early invasive carcinoma, whereas no significant pathological changes were observed in the blank control and solvent control groups.

**Conclusion:**

Endotracheal infusion of Xuanwei bituminous coal dust PM10 induces lung cancer in tree shrews. Thus, the lung cancer model was established.

肺癌在许多国家和地区的发病率和死亡率大幅度增加，成为死亡率最高的恶性肿瘤之一。我国肺癌患者总数已排在世界第一位。云南省的宣威和富源肺癌的发病率和死亡率都很高，其中女性肺癌死亡率居全国首位。是世界上女性肺癌发病率最高的地区。中国预防医学科学院环境卫生与卫生工程研究所就宣威肺癌，展开了多学科的综合性研究，生活燃料与宣威女性肺癌之间存在明显联系。宣威大气中的小于10 μm的颗粒物所占比例远大于其他地方^[1]^。我们前期研究发现：宣威地区居民多采用当地生产烟煤做饭及取暖，燃煤燃烧释放烟尘是宣威地区室内污染主要来源，烟煤粉尘中有磷（P）、钾（K）、钠（Na）、钙（Ca）、铁（Fe）、硅（Si）、锶（Sr）等，并有纳米二氧化硅、苯并芘等致癌物质。含量较非宣威地区烟煤粉尘高。

研究肺癌的发生发展过程及其机制，寻找机体形成实体性肿瘤过程中的早期变化指标就成为预防癌症的重要课题。建立科学合适的肺癌动物模型，用以模拟出与人类肺癌的病因、发病机制、发展过程相似的动物模型是亟待解决的问题。树鼩的许多分子与细胞结构近似于人类，作为接近人类的低等灵长类动物，生理功能与人类接近，适合应用于研究人类疾病的动物模型。Xia等^[2]^发现树鼩是一种很有前途的人类乳腺癌动物模型，本研究探索应用宣威烟煤粉尘行树鼩支气管灌注，通过观察灌注后树鼩一般情况、胸部X线及肺部病理变化，了解树鼩作为肺癌模型动物的可行性。

## 1 材料与方法

### 1.1 实验动物

健康成年树鼩80只（购自中科院昆明动物研究所），随机分为A、B、C组。A组10只，为空白对照组；B组10只，为溶剂对照组；C组60只，为烟尘处理组，C组又随机分为C1、C2、C3、C4四组，每组15只。

### 1.2 主要试剂与仪器

宣威烟煤粉尘（PM10，与合作单位香港中文大学联合收集），碘化油注射液：山海旭东海普药业有限公司，蒸馏水：自备，灌注针：自制。试剂浓度如下：浓度1:0.1 mL混悬液中含：碘化油注射液0.09 mL、烟煤粉尘1 mg+蒸馏水0.01 mL；浓度2:0.1 mL混悬液中含：碘化油注射液0.09 mL、烟煤粉尘2 mg+蒸馏水0.01 mL；浓度3:0.1 mL混悬液中含：碘化油注射液0.08 mL、烟煤粉尘5 mg+蒸馏水0.01 mL；浓度4:0.1 mL混悬液中含：碘化油注射液0.08 mL、烟煤粉尘10 mg+蒸馏水0.01 mL。

### 1.3 实验方法

以氯胺酮100 mg/kg体重注射+阿托品0.67 mg/kg肌肉注射麻醉后，切开颈部皮肤，充分暴露甲状软骨，于甲状软骨上方薄弱处，以特制灌注针行穿刺进入支气管灌注诱癌剂。注射完毕后立即将树鼩直立旋转并轻轻按压其胸部，以使溶剂均匀分布于两肺部；每周注射1次，持续2个月（共灌注8次）。

### 1.4 X线检查

全部动物于药物第一次灌注后第3周、5周、7周、9周、11周，行X线检查，观察是否有肺及胸膜结节，如有结节测量结节大小。并行解剖及病理诊断。

### 1.5 病理检查

X线检查后无异常树鼩于实验3周、5周、7周、9周，随机选A组2只、B组2只、C组2只解剖树鼩，第11周处死所有剩余树鼩，观察有无肿瘤形成，观察胸腔内胸膜、肺、心脏及肋骨内面有无异常改变，如解剖后发现异常，取异常区域组织以10%甲醛溶液固定，无结节者取左肺下叶肺组织以10%甲醛溶液固定，待HE病理检查确定病变性质。实验过程中意外死亡的树鼩行上述步骤处理，并对肿块及肺组织切片进行HE染色分析。具体步骤如下^[3]^：取材与固定：处死动物发现肺内或胸壁及心包结节者取结节部位，无结节者取左肺下叶肺组织，投入10%甲醛溶液进行固定24 h后将组织块取出。组织脱水、透明、浸蜡：80%酒精1 h-90%酒精1 h-95%酒精Ⅰ 1 h-95%酒精Ⅱ 1 h-100%酒精Ⅰ 1 h-100%酒精Ⅱ 1 h-100%酒精Ⅲ 1 h-二甲苯Ⅰ 1 h-二甲苯Ⅱ 1 h-石蜡Ⅰ 1 h-石蜡Ⅱ 1 h-石蜡Ⅲ 1 h（以上步骤均由自动脱水机完成）。包埋：将已透明的肺组织置于已融化的石蜡中，放入溶蜡箱保温，等石蜡完全浸入组织后包埋。切片与贴片：将包埋好的蜡块固定于切片机上，切成5 μm厚的薄片。将薄片放到加热的水中烫平，贴到载玻片上，放入45 ℃恒温箱中烘干。脱蜡染色：将切片放入苏木精水溶液中染色8 min，酸水及氨水中分色各5 s，流水冲洗1 h后放入蒸馏水片刻，放入70%和90%的酒精中脱水各10 min，放入酒精伊红染色液染色3 min。染色后的切片经纯酒精脱水，再经二甲苯透明。已经透明的切片滴上加拿大树胶，盖上盖玻片封固。待胶干后贴上标签，镜下读片^[4]^。

## 2 结果

### 2.1 影像学改变

空白对照组树鼩行X线检查，无明显异常；溶剂对照组树鼩肺部无明显异常显影；烟尘处理组树鼩灌注后肺部药剂灌注部位可见不同程度药剂残留影像，考虑为碘化油注射液未完全代谢所致，经病理切片证实，X线片发现有药剂残留部位肺组织发生支气管上皮改变。（图 1）。

**1:**
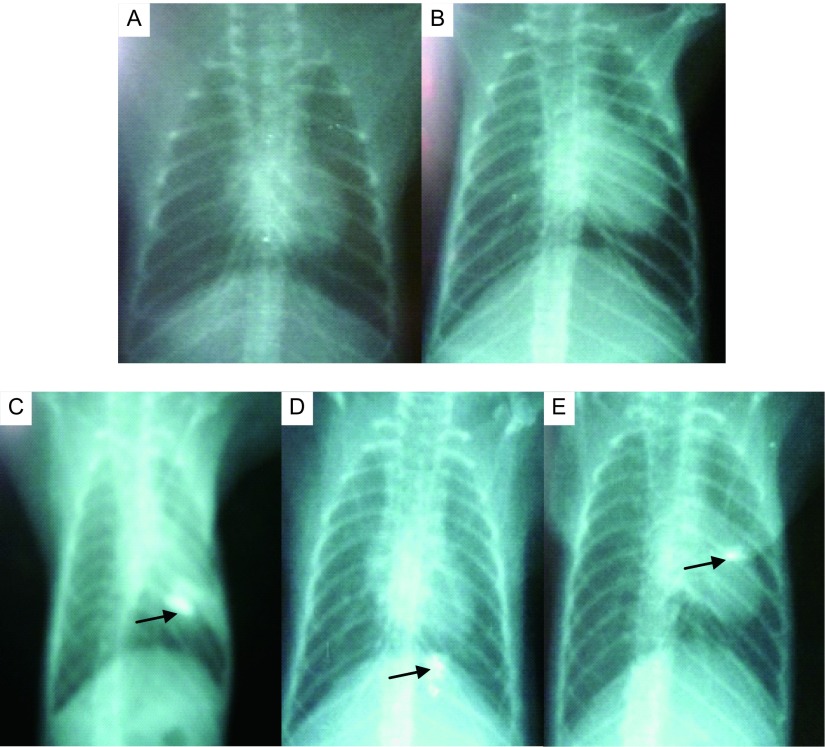
溶剂灌注后树鼩X线检查结果。A：空白对照组；B:溶剂对照组；C:烟尘处理组第3周；D:烟尘处理组第5周；E:烟尘处理组第7周。 Results of X-ray examination after solvent perfusion tree shrew. A: Blank control group; B: Solvent control group; C: The third week of dust treatment group; D: The fifth week of dust treatment group; E: The seventh week of dust treatment group.

### 2.2 病理改变

行肺组织HE染色观察，空白对照组及溶剂对照组肺组织HE染色观察树鼩支气管上皮无明显改变。烟尘处理组第3周处死树鼩肺组织内见充血、炎变、出血及支气管上皮脱落等改变；5周、7周、9周、11周处死树鼩发现支气管上皮复层化、鳞状化生等变化，甚至可见早期浸润癌，支气管上皮改变多伴有炎症出血表现，且随着烟煤粉尘浓度升高，异型增生及癌变率逐渐升高。随支气管灌注时间延长及烟尘浓度增加，树鼩支气管上皮出现支气管粘膜上皮过度增生-鳞状化生-不典型增生-早期浸润癌的病理变化，同时伴有炎症、出血等改变各个阶段变化在同一肺组织切片中可同时存在，以病变程度较高者为数据统计标准。烟尘处理组最早在灌注第3周就发现有轻度-中度不典型增生病变，最早在灌注第7周就发现重度不典型增生，在第9周就有原位癌出现（表 1，图 2）。

**1:** 肺组织病变情况例数统计 The statistics of the pathological changes of lung tissues

Group	Mild dysplasia	Moderate dysplasia	Severe dysplasia	Carcinoma *in situ*	Ther rate of canceration
Blank control group	0	0	0	0	0
Solvent control group	0	0	0	0	0
Dust treatment group (total)	3	6	10	4	13.33%
Concentration 1	1	1	0	0	0
Concentration 2	0	1	3	1	6.67%
Concentration 3	1	2	2	1	6.67%
Concentration 4	1	2	5	2	13.33%

**2:**
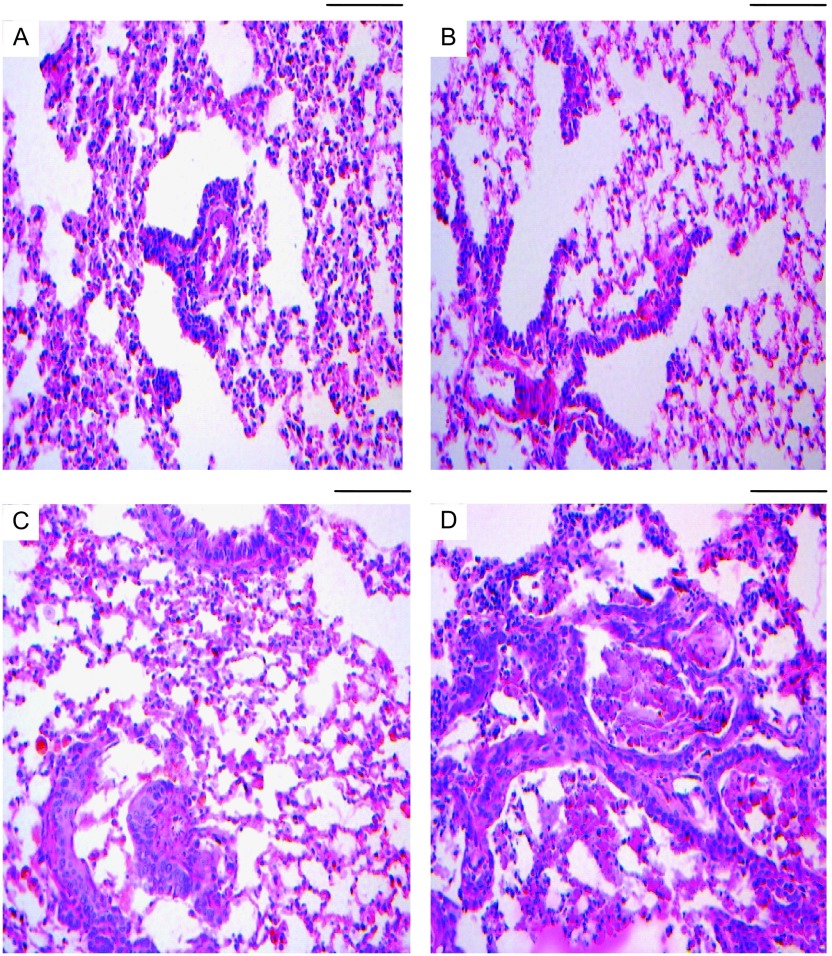
烟尘处理组病理改变。A：轻度不典型增生（HE, ×200）；B：中度不典型增生（HE, ×200）；C：重度不典型增生（HE, ×200）；D：原位癌（HE, ×200）。标尺为0.1 mm。 The pathology change of dust treatment group. A: Mild atypical hyperplasia(HE, ×200); B: Moderate dysplasia (HE, ×200); C: Severe dysplasia (HE, ×200); D: Carcinoma *in situ* (HE, ×200). The ruler is equal to 0.1 mm.

## 3 讨论

建立科学合适的肺癌动物模型，用以模拟出与人类肺癌的病因、发病机制、发展过程相似的动物模型是亟待解决的问题，以此寻求更好的诊断和治疗策略提供有价值的动物模型。一般来说，动物进化程度越高，其结构、功能越复杂，反应也越接近于人类。目前，肺癌动物模型多选择小鼠、大鼠、地鼠、兔、犬和羊等。

已建立的肺癌诱发型动物模型有：用乌拉坦、乙酰胺基氟（AAF）诱发小鼠肺腺癌；用氧化钚诱发大鼠肺腺癌；经支气管灌注碘油、三甲基胆蒽与二乙基亚硝胺混悬液诱发大鼠肺鳞癌；用云锡矿粉（无氡）诱发F344大鼠诱发肺鳞癌、腺癌^[5]^；用苯并芘成功诱发小鼠形成原发性肺癌^[6]^。诱发性肺癌动物模型发生的条件比较自然，与人类患病过程相似。常用的移植性肺癌动物模型有：①将肺癌细胞接种于裸鼠或其他免疫缺陷动物的皮下；②将小鼠的气管分离，经过反复冻融处理，然后将肺癌细胞接种到气管，结扎气管两端后，再将气管移植到重度复合免疫缺陷小鼠的皮下^[7]^；③切开胸壁皮肤，将肺癌细胞或肺癌组织直接种植到肺脏内^[8]^。综合以上已经建立的肺癌动物模型，虽然在各个方面都能较好地模拟人类肺癌，但是由于大多选用啮齿类动物，虽然其在组织、形态、分子特点上与人类比较相近，但其呼吸道结构及气管上皮等结构与人类有明显差异，不能很好地模拟人类肺癌。

树鼩，攀鼩目，树鼩科，具有一些与原始灵长类的近似特征，部分灵长类学者相信，树鼩不失为研究祖先灵长类的模特，或者把树鼩当做连接食虫类和灵长类的居间类群。现已成功建立了柯萨奇病毒A16等多种人类病毒感染的动物模型^[9]^，并在神经生物学、生殖生物学、免疫学和社会心理学等方面有相当广泛和深入的应用和研究^[10, 11]^。Park等^[12]^对树鼩肝组织的野生型p53进行分析后发现，树鼩p53与人类p53的核酸序列同源性以及氨基酸序列同源性分别高达91.17%和93.14%，而与小鼠p53的同源性则分别仅为77.12%和73.7%。谢丽等^[13]^对实验室繁育的22只成年树鼩进行生理指标的检测，并与正常人参考值进行对比分析。结果显示，树鼩红细胞数8.19×10^12^/L，高于人参考值上限；白细胞数2.21×10^9^/L，低于人参考值下限；肌酐16.63 μmol/L，低于人参考值下限；丙氨酸氨基转移酶77.92 U/L和天门冬氨酸氨基转移酶179.9 U/L，均高于人参考值上限。说明树鼩作为接近人类的低等灵长类动物，生理功能与人类接近，适合应用于研究人类疾病的动物模型。而且个体小、易操作、对环境的适应性好、繁殖快，易驯养和饲育，新陈代谢更接近于人，实验成本低。目前树鼩主要用于肝炎病毒、肝癌、近视和社会心理应激模型的构建^[14]^。探索建立树鼩肺癌动物模型，将在更大程度上模拟人类肺癌的发生发展过程，为进一步研究人类肺癌的发病提供良好的实验动物。

大鼠肺癌模型其气道上皮发展为侵袭性鳞癌的形态学发展顺序是：上皮细胞增生-鳞状上皮化生及异常增生-原位癌，原位鳞癌可能不一定有上皮层增厚，但可见核分裂相和异型性显著，鳞状上皮分化成熟层次消失^[15]^。用宣威烟煤粉尘灌注大鼠支气管，其诱发病变过程完全符合气道上皮发展为侵袭性鳞癌的形态学变化，灌注后依次出现支气管粘膜上皮过度增生-鳞状化生-不典型增生-早期浸润癌-广泛浸润的高分化鳞癌的发展过程，而且所有诱发性肺癌均为高分化及低分化鳞状细胞癌，这种改良型肺癌动物模型诱癌成功率高、成本低、诱癌时间短，作为单因子诱发的实验动物肺癌模型，对于进一步研究致癌机制、肿瘤免疫以及抗癌阻断治疗，提供了较为适宜的肺癌模型手段^[16]^。

一项实验^[17]^研究了多种PM样本如总悬浮颗粒物（total suspended particulate, TSP）、PM10、PM2.5、柴油尾气微粒、汽油尾气微粒和木柴燃烧的烟雾等产生（·OH）的情况，结果显示，PM产生（·OH）的能力与水溶性金属的浓度有关，尤其是与铁（Fe）、钒（V）和其他离子化转换的金属有关，而不是与总的金属含量有关。金属离子除了直接作用外，还可以通过改变其他物质的性质而起作用。林春芳等^[18]^发现多种金属阳离子（如铁、铝、镁、钛）等均可以与石英粉尘表面的硅烷醇基（-SiOH）络合，改变石英粉尘的离子化状态和产生自由基的能力，从而影响石英粉尘的毒性，由亚铁离子催化生成的自由基是石英粉尘致癌的一个主要因素。

本实验采用树鼩为实验动物，参考大鼠诱发肺癌动物模型的构建，发现树鼩在应用宣威烟煤粉尘行支气管灌注之后，其胸部X线检查无明显改变，但灌注部位肺组织行HE染色病理检查发现，支气管粘膜上皮过度增生-鳞状化生-不典型增生-早期浸润癌的病理变化过程，考虑用此种方法可模拟原发肺癌发生发展过程。本实验中可发现支气管上皮复层化、鳞状化生等变化，甚至可见早期浸润癌，但是X检查无见肿瘤，病理发现早期浸润癌实验动物例数较少，多为支气管上皮复层化及鳞状上皮化生改变，考虑可能与实验时间过短影响，若实验观察时间及处死时间延长可能会获得更为满意的结果。
